# High ovarian responders have the highest risk of premature progesterone rise

**DOI:** 10.5935/1518-0557.20240004

**Published:** 2024

**Authors:** Alfredo Cortés-Vazquez, Greys Thelma Vásquez-Ramírez, Alfredo Leonardo Cortés-Algara, Jesús-Daniel Moreno-García, Panagiotis Drakopoulos

**Affiliations:** 1 Reproductive Endocrinology Specialist, Centro Médico Nacional 20 de Noviembre, Mexico City, Mexico; 2 Associate Professor, Obstetrics and Gynecology Department, Escuela Superior de Medicina, Instituto Politécnico Nacional, Mexico City, Mexico; 3 Reproductive Endocrinology Fellow at Reproductive Endocrinology Department, Centro Médico Nacional 20 de Noviembre, Mexico City, Mexico; 4 Minimally Invasive Gynecological Surgery Department, Centro Médico Nacional 20 de Noviembre, Mexico City, Mexico; 5 Reproductive Endocrinology Professor at Universidad Nacional Autónoma de México; 6 Reproductive Endocrinology Specialist, Reproductive Endocrinology Department, Centro Médico Nacional 20 de Noviembre, Mexico City, Mexico; 7 Centre for Reproductive Medicine, Universitair Ziekenhuis Brussel, Vrije Universiteit Brussel, Brussels, Belgium; 8 Department of Obstetrics and Gynaecology, School of Medicine, European University Cyprus, Nicosia, Cyprus; 9 IVF Athens, Athens, Greece

**Keywords:** risk, premature, progesterone, ovarian, response

## Abstract

**Objective:**

Late follicular phase progesterone elevation is a complication that affects approximately 38% of IVF cycles. There is a lack of consensus on the appropriate cut-off levels for progesterone on hCG day. Although premature progesterone rise occurs in all kinds of ovarian responses, there is a knowledge gap regarding the ovarian response with the highest risk of this phenomenon. Our study aims to assess the relative risk of each kind of ovarian response for premature progesterone rise and evaluate the prevalence of premature progesterone rise in each ovarian response.

**Methods:**

A retrospective, cross-sectional, comparative and analytic study was performed at the Reproductive Endocrinology Department in Centro Médico Nacional 20 de Noviembre in Mexico City. All conventional-antagonist cycles were grouped according to their ovarian response and were evaluated from 2015 to 2020. Pearson’s Squared-chi, Cramer’s V, cross-table and the relative risk were calculated.

**Results:**

The prevalence of premature progesterone rise oscillated from 20.8 to 67.9% for low and high ovarian responders, respectively. After calculating the relative risk, high ovarian responders had a 1.38 higher risk for premature progesterone rise than other groups.

**Conclusions:**

High ovarian responders have the highest risk for premature progesterone rise compared to normal and low ovarian responders. High ovarian responders have a 67.9% prevalence of premature progesterone rise.

## INTRODUCTION

Late follicular phase progesterone elevation is a complex phenomenon affecting 12 to 38% of in-vitro fertilization (IVF) cycles (Drakopoulos *et al*., 2019). Until today there is a lack of consensus on the optimal cut-off levels for progesterone on hCG day, ranging between 0.8 to 2.0 ng/ml. While the most commonly used cut-off level is 1.5 ng/ml, as suggested by some authors (Bosch *et al*., 2010). There is also evidence that progesterone levels on hCG day have limited regulation since lower progesterone levels also affect live-birth rates (Arvis *et al*., 2019).

Diverse authors have addressed the deleterious effects of premature progesterone elevation on endometrial receptivity and its impact on pregnancy rates after a fresh embryo transfer (Van Vaerenbergh *et al*., 2011; Drakopoulos *et al*., 2019; Xiong *et al*., 2020). However, the aetiology of late follicular phase progesterone elevation is still debatable; some authors consider that several mechanisms can interact in the same patient (Cortés-Vazquez *et al.,* 2021; 2022). Some authors have found that ovarian response to stimulation protocols is strongly related to progesterone levels on hCG day (Sonigo *et al*., 2014). Using data from a large prospective randomized controlled trial, Yding Andersen *et al*. (2011) demonstrated that late follicular phase progesterone is governed by the number of preovulatory follicles and LH concentrations. To the best of our knowledge, no studies are addressing the risk for premature progesterone rise based on the ovarian response. This retrospective study assesses the relative risk for premature progesterone rise in every ovarian response.

## MATERIAL AND METHODS

### Study population

We performed a retrospective analytic and comparative study; all IVF/ICSI cycles performed in the Reproductive Endocrinology Department at Centro Médico Nacional 20 de Noviembre from 2015 to 2020 were analysed. Notably, the Ethics Committee (Institutional Board Review) from Centro Médico Nacional 20 de Noviembre approved this study. All conventional/ antagonist ovarian cycles were included. Patients were excluded if they had a double ovarian stimulation cycle, progestin-primed ovarian stimulation, mild ovarian stimulation cycles, incomplete information and fertilitypreserving cycles for oncologic reasons. From all cycles, age, body mass index, estradiol, progesterone on hCG day, stimulation length, number of preovulatory follicles, total FSH dosage and number of oocytes retrieved were recorded. All patients were stimulated as previously described (Cortés-Vazquez *et al*., 2021).

A convenience-sampling method was performed, and 563 cycles were reviewed and analyzed. All cycles were classified according to their ovarian response. Cycles were classified according to Bologna Criteria to define a poor ovarian response. Group 1 had less or equal to 3 oocytes retrieved, group 2 had 4 to 7 oocytes retrieved, Group 3 had 8 to 14 oocytes, and Group 4 had more than 15 retrieved oocytes. Premature progesterone rise was defined by a serum progesterone value of more than 0.9 ng/ml progesterone level on hCG day.

### Statistical analysis

A Kolmogorov-Smirnov test was performed to evaluate the data for normality. Subsequently, a cross table was generated, and a Pearson’s Chi-squared test was performed to compare the risk of premature progesterone rise according to ovarian response. Afterwards, a Cramer’s V test was used to estimate the effect size. Finally, the relative risk was calculated for each kind of ovarian response. Statistical analysis was performed using SPSS version 25 (IBM), and *p*<.05 was considered statistically significant.

## RESULTS

The primary dataset included 563 cycles from 2015 to 2020; after applying inclusion and exclusion criteria, 530 cycles were eligible for analysis. At Table 1, we summarized our population’s characteristics. Statistically significant differences between groups in the number of follicles >14mm, number of retrieved oocytes, progesterone-per-follicle index, oestradiol and progesterone on hCG day were found (Table 2). Although age was significantly different between groups, we assume it is clinically irrelevant. Notably, there was no difference in total FSH dosage among groups, and the high responders had the highest progesterone level on hCG day. We found that the high responders had the most elevated risk for premature progesterone rise (Table 3). Pearson’s Chi-squared test showed a *p*<.000, and Cramer’s V showed a medium effect size (Tables 4 and 5). The prevalence of elevated progesterone levels on hCG day in high responders was 67.9%, while in other groups ranged from 20.8 to 47.2%. We calculated the relative risk for each group; as shown in Table 6, high-responders had the highest relative risk for premature progesterone rise, with a 1.38 RR. Relative risks according to the type of ovarian response can be found in Table 6.

**Table 1:** Demographic and clinical data.

	Mean	SD
Age	35.7	±3.5
BMI	25.9	±3.6
Stimulation Length	9.9	±1.5
Total FSH dosage (IU)	2439.1	±943.9
No. of follicles >14 mm	8.7	±5.2
Progesterone on hCG day (ng/ ml)	1.13	±1.32
Oestradiol on hCG day (pg/ml)	2679.46	±5358.39
No. of oocytes	6.25	±4.69
Progesterone-per-follicle index	0.16422	±0.18695

hCG= human chorionic gonadotrophin, FSH = follicle stimulating hormone, BMI= Body Mass Index, SD = standard deviation.

* Values presented as mean± standard deviation

**Table 2:** Comparison of groups.

	Low ovarian responders	Suboptimal response	Normal ovarian response	High-ovarian response	p-Value
Age	36.5±3.7	35.7±3.4	35±3.1	34.5±3.9	*p*<0.000
BMI	25.5±3.7	26.2±3.5	26.2±3.1	25.3±3.6	NS
Stimulation Length	9.9±1.7	9.8±1.5	10±1.3	10.3±1.9	NS
Total FSH dosage	2579.91±1199.30	2398.91±811.81	2313.86±766.13	2518.75±792.92	NS
No. of follicles >14mm	4.7±3.03	8.4±3.55	12.4±4.8	16.6±5.8	*p*<0.000
Progesterone on hCG day (ng/ml)	.824±.808	1.06±1.163	1.42±1.53	2.00±2.48	*p*<0.000
Oestradiol on hCG day (pg/ ml)	1232.46±2361.94	2041.42±2078.43	3797.50±6405.02	9838.56±14245.13	*p*<0.000
No. of oocytes	1.75±1.04	5.25±1.11	10.34±1.79	18.75±3.65	*p*<0.000
Progesteroneper-follicle index	0.228±.2402	0.141±0.1450	0.128±0.1547	0.1169±0.1253	*p*<0.000

NS= not significant

* Values presented as mean±standard deviation hCG= human chorionic gonadotrophin FSH= follicle stimulating hormone.

**Table 3:** Cross-Table.

		Premature progesterone rise	
		No	Yes
**Type of ovarian response**	Low-ovarian response	n=133 / 79.2% 4.6^**^	n=35 / 20.8% -4.6^**^
	Suboptimal response	n=128 / 67.4% 0.8^**^	n=62 / 32.6% -0.8^**^
	Normal ovarian response	n=76 / 52.5% -3.7^**^	n=68 / 47.2% 3.7^**^
	High-ovarian response	n=9 / 32.1% -3.8^**^	n=19 / 67.9% 3.8^**^

** Adjusted residuals.

**Table 4:** Chi-squared test.

	Value	p-Value
Pearson’s Chi-squared test	38.15	0.000

**Table 5:** Symmetric Measures.

	Value
Phi	0.268
Cramer’s V	0.268

** *p*-Value <0.000.

**Table 6:** Premature progesterone risk according to ovarian response.

Type of ovarian response	Relative Risk
Low-ovarian response	.29 RR
Suboptimal ovarian response	.58 RR
Normal ovarian response	1.09 RR
High-ovarian response	1.38 RR

## DISCUSSION

To the best of our knowledge, this is the first study to assess the relative risk for each ovarian response to develop premature progesterone rise. In our high-responder population, premature progesterone rise prevalence was surprisingly high compared to the 30.2% prevalence reported in other studies (Requena *et al*., 2014). Racial and ethnic differences between populations could explain this higher prevalence. In a retrospective cohort study, some authors observed that serum progesterone on the hCG day was higher in Latino and Asian women than in white women (Hill *et al*., 2017). Several authors have shown similar results, particularly that increased follicular development and the number of oocytes retrieved are associated with higher progesterone levels on hCG day (Ubaldi *et al*., 1995; Yding Andersen *et al*., 2011; Requena *et al*., 2014; Racca *et al*., 2018; Oktem *et al*., 2019). On the contrary, other authors reached different conclusions, finding that there were no significant differences regarding the number of retrieved oocytes in patients with or without high progesterone levels (Fanchin *et al*., 1997; Kofinas *et al*., 2016).

Another finding in our study was that patients with high progesterone levels on hCG day do not have higher gonadotropin consumption compared to normal or lower responders, which is coincident with other publications (Yding Andersen *et al*., 2011; Lee *et al*., 2014; Arvis *et al.*, 2019; Oktem *et al*., 2019). However, other investigators have found an association between elevated preovulatory progesterone levels and higher gonadotropin consumption (Ubaldi *et al*., 1995; Fanchin *et al*., 1997; Kofinas *et al*., 2016; Cao *et al*., 2020).

Our results are relevant, above all, for patient counselling and treatment individualization. Higher responders have a higher probability of live birth after a freezeonly cycle strategy irrespective of progesterone concentration compared to fresh embryo transfer (Bosdou *et al*., 2019; Yu *et al*., 2020). So it would be reasonable to implement a freeze-all policy in high ovarian responders since the risk of ovarian hyperstimulation syndrome (OHSS) would be abolished, along with the premature progesterone rise risk. Stormlund *et al*. (2019) demonstrated that nearly 60% of patients would prefer a freeze-all strategy where the pregnancy outcome was equal to fresh embryo transfer. Approximately 90% of infertile women would opt for a frozen embryo transfer if ovarian stimulation implies any risk to the mother or child (Stormlund *et al*., 2019). The freeze-all strategy would also allow physicians to use a more patient-friendly in high-responders, with progestin-primed ovarian stimulation without compromising oocyte or embryo safety, as shown by Zhu *et al*. (2021); albeit more evidence is needed.

## CONCLUSIONS

High responders have the highest risk among all the ovarian responses. Physicians should assess patients about the increased risk of premature progesterone rise and consider implementing a freeze-all policy in high responders.
